# Deodorants and antiperspirants: New trends in their active agents and testing methods

**DOI:** 10.1111/ics.12852

**Published:** 2023-03-21

**Authors:** Paweenuch Teerasumran, Eirini Velliou, Shuo Bai, Qiong Cai

**Affiliations:** ^1^ Department of Chemical and Process Engineering, Faculty of Engineering and Physical Sciences University of Surrey Guildford GU2 7XH UK; ^2^ Centre for 3D Models of Health and Disease UCL‐Division of Surgery and Interventional Science Charles Bell House, 43‐45 Foley Street, Fitzrovia London W1W 7TY UK; ^3^ The State Key Laboratory of Biochemical Engineering, Institute of Process Engineering Chinese Academy of Sciences Beijing 100190 China

**Keywords:** antiperspirant, body odour, delivery/vectorization/penetration, deodorant, formulation/stability, safety testing, sweat glands, sweating

## Abstract

Sweating is the human body's thermoregulation system but also results in unpleasant body odour which can diminish the self‐confidence of people. There has been continued research in finding solutions to reduce both sweating and body odour. Sweating is a result of increased sweat flow and malodour results from certain bacteria and ecological factors such as eating habits. Research on deodorant development focuses on inhibiting the growth of malodour‐forming bacteria using antimicrobial agents, whereas research on antiperspirant synthesis focuses on technologies reducing the sweat flow, which not only reduces body odour but also improves people's appearance. Antiperspirant's technology is based on the use of aluminium salts which can form a gel plug at sweat pores, obstructing the sweat fluid from arising onto the skin surface. In this paper, we perform a systematic review on the recent progress in the development of novel antiperspirant and deodorant active ingredients that are alcohol‐free, paraben‐free, and naturally derived. Several studies have been reported on the alternative class of actives that can potentially be used for antiperspirant and body odour treatment including deodorizing fabric, bacterial, and plant extracts. However, a significant challenge is to understand how the gel‐plugs of antiperspirant actives are formed in sweat pores and how to deliver long‐lasting antiperspirant and deodorant benefits.

## Graphical abstract

This article is a systematic review of recent progress in developing novel antiperspirant and deodorant active ingredients. Furthermore, this article summarized the development of the testing platform used to evaluate the mechanism of action and the efficiency of those products.

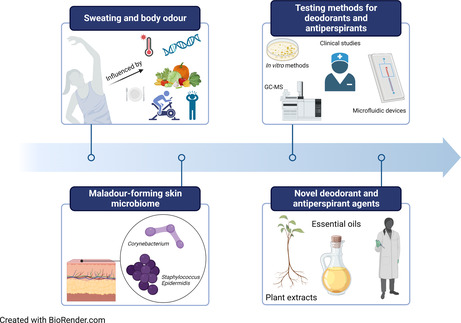

## Résumé

La transpiration est le système de thermorégulation de l’organisme, mais elle entraîne également une odeur corporelle désagréable qui peut diminuer la confiance en soi. Des nombreuses recherches ont été menées afin de trouver des solutions pour réduire à la fois la transpiration et l’odeur corporelle. La transpiration est le résultat de l’augmentation du flux de sueur, et les mauvaises odeurs sont dues à certaines bactéries et à certains facteurs écologiques tels que les habitudes alimentaires. Les recherches sur le développement des déodorants se concentrent sur l’inhibition de la croissance des bactéries responsables des mauvaises odeurs à l’aide d’agents antimicrobiens, tandis que les recherches sur la synthèse des anti‐transpirants se concentrent sur les technologies diminuant le flux de sueur, ce qui réduire non seulement les odeurs corporelles, mais améliore également l’apparence des personnes. La technologie des anti‐transpirants repose sur l’utilisation de sels d’aluminium qui peuvent former un bouchon de gel au niveau des pores sudoripares, empêchant le liquide sudoral d’apparaître à la surface de la peau. Dans cet article, nous effectuons une revue systématique des progrès récents réalisés dans le développement de nouveaux principes actifs anti‐transpirants et déodorants qui sont sans alcool, sans parabène et d’origine naturelle. Plusieurs études ont été rapportées sur la classe alternative de principes actifs qui peuvent potentiellement être utilisés pour le traitement anti‐transpirant et des odeurs corporelles, y compris les tissus désodorisants, les bactéries et les extraits végétaux. Cependant, un défi important consiste à comprendre comment les bouchons de gel des actifs anti‐transpirants se forment au niveau des pores sudoripares, et comment offrir des effets anti‐transpirants et déodorants durables.

## INTRODUCTION

Sweating is a physiological function of the human body to regulate the body's core temperature which can be triggered by stress, anxiety, and diet [1]. Initially, sweat is an odourless liquid mostly comprised of water, electrolytes, and proteins. The formation of an unpleasant odour is a result of the metabolic activity of human natural microflora habituated in the axillary region. Additionally, the body odour intensity and chemical composition can be influenced by various factors including gender, dietary intake, age, and race [2, 3].

Sweating and body odour can have a significant negative effect on people's appearance, confidence, and may be viewed as a sign of poor hygiene [4]. Therefore, there is a continued research interest in developing topical deodorants and antiperspirants and the sector generates over $1.5 billion annual revenue [5, 6]. Products on the market are still based on a group of well‐known and established active ingredients for both deodorant and antiperspirant introduced over 50 years ago [7]. As the skin microbiome is the cause of body odour, the mechanism of action of deodorant products is to inhibit the growth of malodour bacteria by using antimicrobial bacteriostatic agents such as Triclosan. Furthermore, perfumes, fragrances, and essential oils are added into a deodorant formulation in order to mask the unpleasant body odour. For antiperspirants, their mechanism of action is relying on the use of aluminium salts such as aluminium chloride which has been developed since 1916.

Over the recent years, consumers and researchers alike has grown an increasing interest to search for alternative actives that are alcohol‐free, paraben‐free, and naturally derived to deliver deodorant and antiperspirant benefits. Much of the challenge is in understanding the gelation mechanism (the ability to form a gel that physically block the sweat pore opening) and the dose response of antiperspirants in the sweat pore's environment. This article presents a review of the current progress in research on understanding body odour treatment and the mode of action of metallic salts. It then discusses the research progress of naturally derived body odour treatment agents as an alternative to the metallic salts and existing chemical compounds.

## SWEAT GLANDS AND THEIR FUNCTIONS

### Eccrine, apocrine, and apoeccrine glands

**FIGURE 1:**
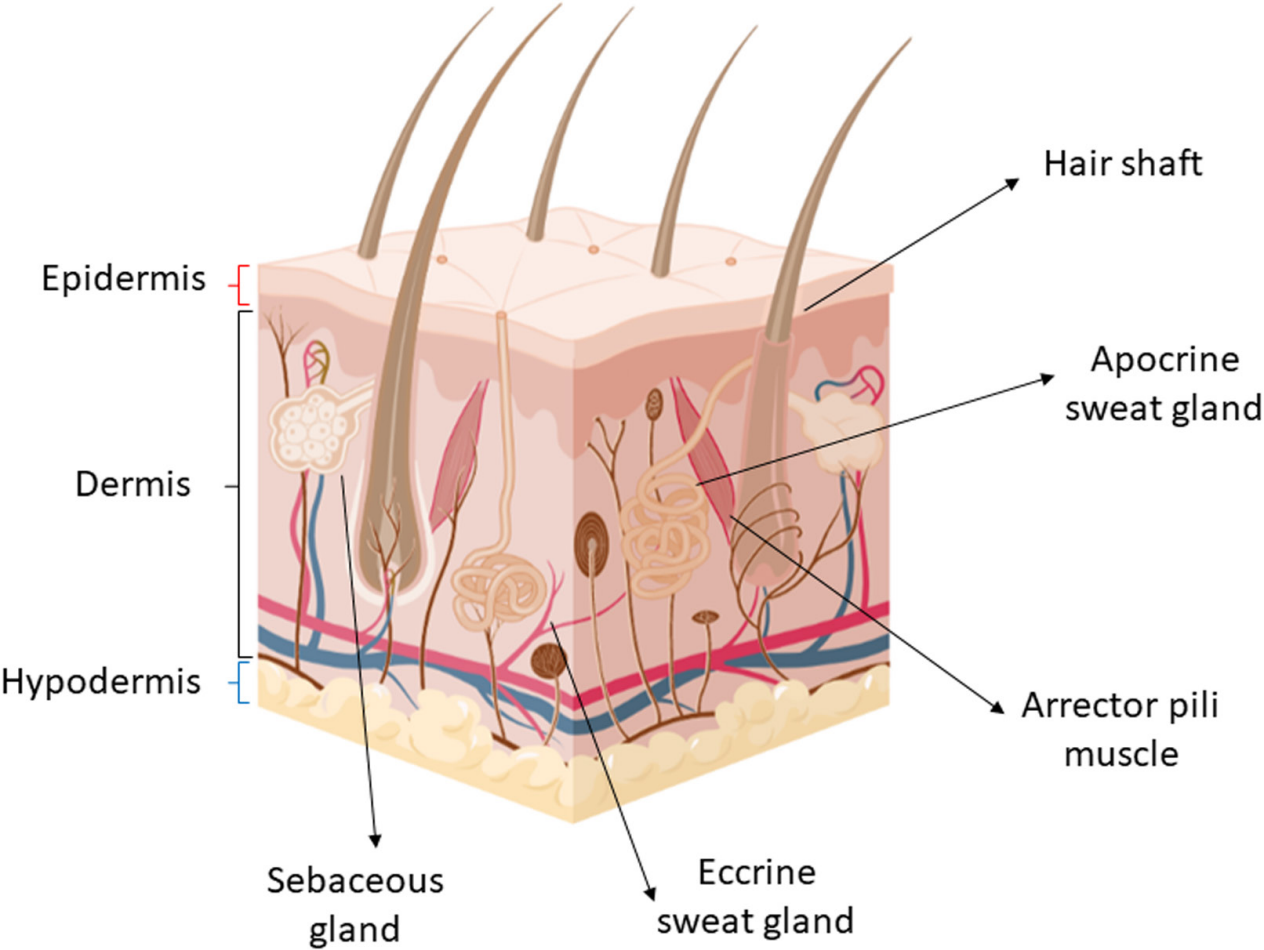
Schematic illustration of human skin structures and skin appendages (Created with BioRender.com).

Skin is a part of the human integumentary system which forms the outermost layer of the human body. Skin is the largest organ in the body which provides a mechanical barrier, protecting the body from the external environment [8]. In addition, skin also contributed to both endocrine and exocrine functions of the human body. Examples of an exocrine function of the skin are secretion of sebum and sweat fluid. There are three main compartments of human skin, epidermis, dermis, and the subcutaneous fascia. Sweat glands are skin appendages found at the dermis compartment of the human skin [8, 9]. It was estimated that there is a total of 2 380 000 glands distributed all over the body [10]. An early morphological study in 1917 showed that there are two types of sweat glands, eccrine and apocrine sweat glands (illustrated in Figure 1) [11].

Eccrine sweat glands are distributed all over the body except glans penises and lips [12]. Eccrine glands play a critical role in the human body's thermoregulation system [2]. The sweat fluid secreted from eccrine glands is a watery fluid with inorganic salts such as NaCl, KCl, and organic acids, including lactic acid and urea. Additionally, antimicrobial peptides such as immunoglobulins and dermcidin are found in trace amount in the eccrine secreted fluid [13]. The perspiration from eccrine glands is emptied directly onto the skin surface [14]. The duct opening of eccrine sweat glands is located at the epidermal compartment of the skin and referred to as the acrosyringium. The luminal diameter of the acrosyringium is between 20 and 60 μm [15].

Apocrine sweat glands can only be found in a certain area of the body, for instance, genital, plantar, and axillary. In contrast to eccrine glands, apocrine glands start functioning at puberty. Apocrine glands are roughly 800 μm larger than eccrine glands and their ducts open to the hair follicle [14]. The fluid secreted from apocrine glands is oily, consisting of fatty acids, proteins, and steroids [16]. However, this fluid is mixed with the sebum (secreted from the sebaceous gland) as both glands are opened into the hair follicle.

A study conducted by Sato et al. [17] has introduced the third type of sweat gland, the apoeccrine gland. Apoeccrine glands are described as having a seven‐fold higher sweat secretion rate than eccrine glands. They are developed during puberty from an eccrine‐like precursor and contributed up to 45% of sweat glands found in the axillary region. However, another study by Bovell in 2007 has debunked that such type of sweat gland cannot be found [14].

### Hyperhidrosis

Hyperhidrosis is referred to as a disorder characterized by over‐secretion of sweat beyond the physiologically required amount, especially at the axilla [4]. About 2%–3% of the population suffers from this condition. The possible cause of hyperhidrosis is the abnormality in sympathetic and parasympathetic nervous activities, overstimulating the sweat gland. Due to such an excessive amount of water, hyperhidrosis is frequently accompanied by bromhidrosis (foul‐smelling body odour). Hyperhidrosis may not only cause inconvenience such as changing drenched clothes and shoes but also can affect the patient's psychological wellbeing [18]. To adequately treat hyperhidrosis, a medicated antiperspirant with a much higher concentration of aluminium salts than a commercially available product is used.

## RESEARCH PROGRESS OF DEODORANTS

### Body odour formation mechanism and its chemical composition

Skin microbiomes are responsible for the production of human body odour [4, 5]. Sweat secreted from sweat glands is initially an odourless fluid. Once the fluid reaches the skin surface, its biomolecule components are being digested by the skin microbiome, generating volatile malodourous compounds. An axillary region is an excellent place for the skin microbiome to proliferate due to its warm and moist environment covered with dense hair follicles. Additionally, the human axilla has a high density of both eccrine and apocrine sweat glands (more than 25 000 in total for each axilla) [19]. There are four primary types of bacterial species involving in malodour compound production, Propionibacterium, Micrococci, Staphylococcus, and Corynebacterium with the latter two being the most prominent [20]. Additionally, the diversity of natural microflora species is distinctive to each individual [21].

**FIGURE 2:**
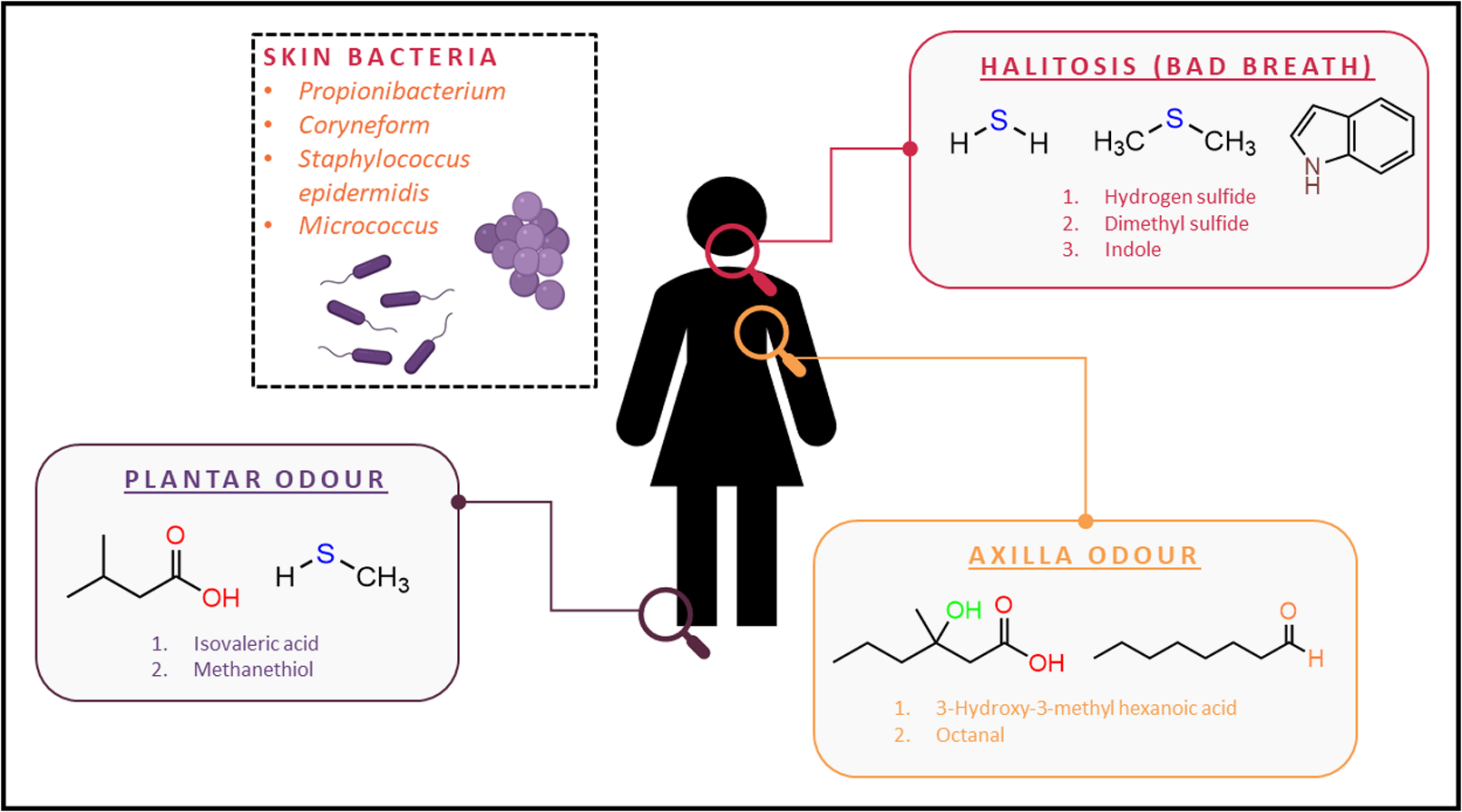
Schematic representation of chemical components in human body odour [Created with BioRender.com, inspired by 24, 26].

The threshold for body odour perception can be varied from one individual to another. Sometimes, body odour is unnoticeable to an individual as that person may develop specific anosmia [22]. When the odour is pointed out, they may feel enormously embarrassed. In modern‐day life, both sweating and body odour are not only affecting the first impression and self‐confidence level of an individual but also leading to economic consequences such as replacing stained clothes and shoes. Figure 2 summarized the chemical components of the human body odour.

Aldehydes are recognized to be one of the major constituents in axilla odour [23]. Over 85%–89% detection rate of aldehydes (octanal, nonanal, and decanal) from human axilla odour using the headspace gas chromatography/mass spectrometry was reported by Haze et al. [24]. In addition to aldehydes, sulphur and carboxylic acid are odorants produced at the axilla [24]. The presence of malodour sulphur compound, (S)‐3‐Methyl‐3‐Sulfanylhexan‐1‐ol (3M3SH) was reported by Starkenmann et al. in 2005 and Bawdon et al. in 2015 [25, 26]. The findings from both studies suggested that *Staphylococcus hominis*, *Staphylococcus lugdunesis*, and *Staphylococcus haemolyticus* were responsible for the formation of 3M3SH from a cysteine–glycine (Cys‐Gly) conjugate precursor. 3M3SH is a malodour substance with extremely high sensitivity in humans with a threshold of 0.001 ng/L air (reported by Natsch et al. 2004) [27]. A possible reason for such low detection threshold is their high affinity to human olfactory receptor OR2M3 [28].

**FIGURE 3:**
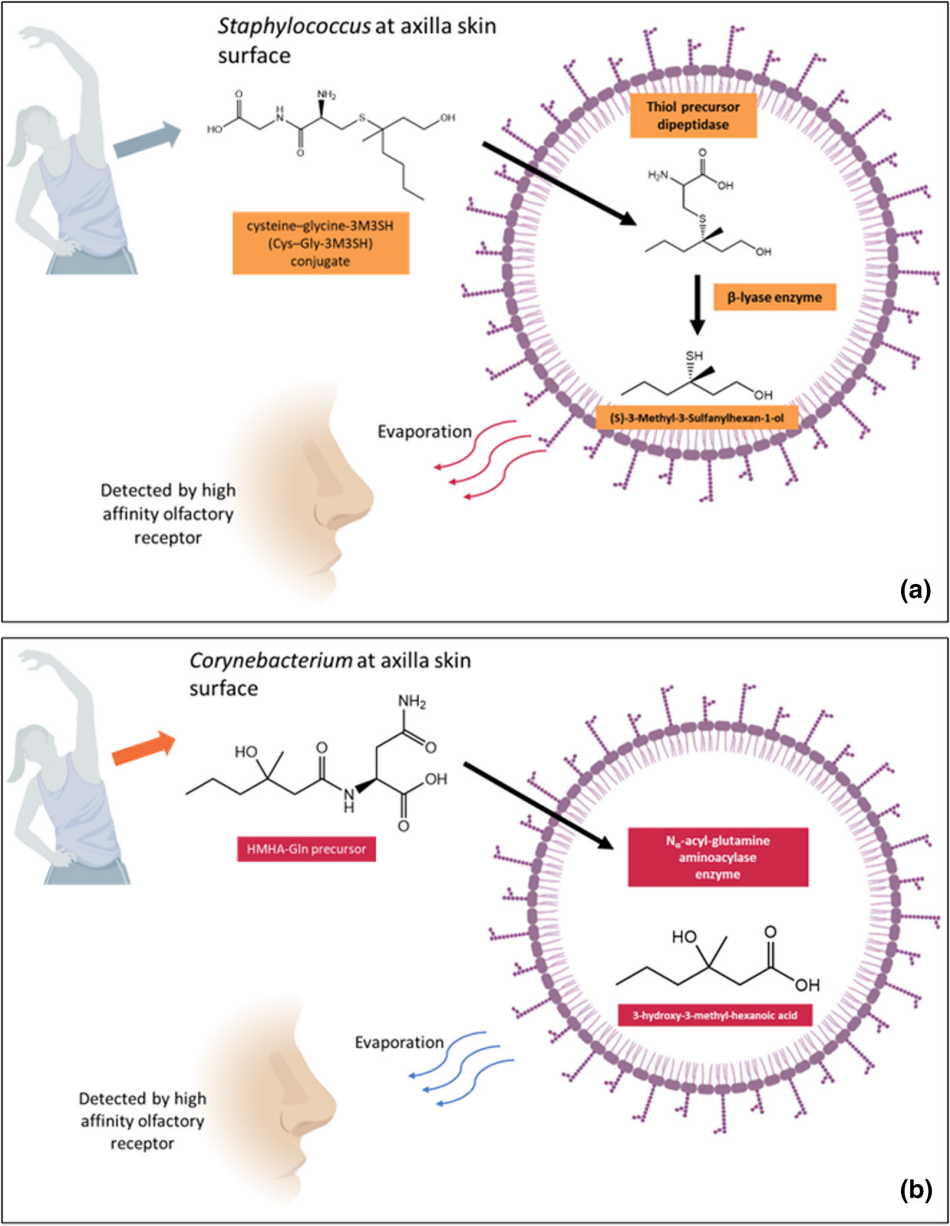
(a) Formation mechanism of (S)‐3‐Methyl‐3‐Sulfanylhexan‐1‐ol (3M3SH) and (b) 3‐hydroxy‐3‐methyl‐hexanoic acid (HMHA), created by Biorender.com [78].

Additionally, 3‐hydroxy‐3‐methyl‐hexanoic acid (HMHA) is another common malodour compound with high sensitivity found at the axilla with a threshold of 0.0044 ng/L air. Figure 3a,b summarized the formation mechanism of 3M3SH by *Staphylococcus* bacteria, and HMHA by *Corynebacterium* at the axilla, respectively.

Various factors can affect the chemical composition and the intensity of body odour, for instance, race, gender, climate, diet, age, as well as behavioural habits such as drinking and smoking [23, 29]. Interestingly, several studies have shown that a mutation of ABCC11 protein in the Asian population leads to a reduced concentration of body odour precursor [30]. Another example is the presence of age‐dependent unsaturated aldehyde, 2‐Nonenal. A clinical study conducted by Haze et al. [24] showed that 2‐nonenal was only detected in subjects who were 40 years old or older.

### Deodorant's ingredients

The mechanism of action of deodorants is relying on the use of antimicrobial agents to inhibit the growth of body odour‐forming microbiome. Additionally, perfume, fragrance, and essential oils are often found in a deodorant formulation to mask the body odour. Ingredients of commercially available deodorant products are summarized in Table 1 [31].

**TABLE 1:** Common ingredients in deodorant products, adapted from Martini 2020 [31].

Role	Compound
Antimicrobial agents	Triclosan, propylene glycol, quaternary ammonium compounds, octoxyglycerin, 2‐ethylhexylglycerin, and ethyllauryl arginate hydroxychloride
Odour masking agents	Limonene, linalool, eugenol, geraniol, hexylcinnamaldehyde, or simply labelled “fragrance”
Odour absorbers	Sodium acid carbonate, zinc carbonate, and talc

There are three essential ingredients within a deodorant formulation: (1) antimicrobial agents, (2) fragrance, and (3) odour absorbers. Triclosan (5‐chloro‐2‐(2,4‐dichlorophenoxy)phenol) is one of the most widely used anti‐microbial agents introduced since 1967. It is a non‐ionic antimicrobial agent which provides good stability and a lack of resistance among odour production bacteria [32]. In addition to triclosan, quaternary ammonium compounds such as benzethonium chloride, the vehicle of the deodorant product (for example, propylene glycol), and sodium bicarbonate (baking soda, NaHCO_3_) are also found to have an antimicrobial activity [33]. Nonetheless, complete suppression of human natural microflora can potentially lead to skin vulnerability [34].

Acidifiers were introduced in the 1990s to reduce the local pH to inhibit the growth of bacteria at the axilla [35]. Since then, several studies have shown that reduction in skin surface pH can lessen the bacteria count [36, 37]. For example, Lukacs and colleagues (1991) studied the pH effect on the growth of bacterium and introduced triethylcitrate into deodorant formulation to reduce the local pH at the axilla. However, triethylcitrate did not successfully convert to citric acid after the application and the pH remains unchanged [35]. Later in 2000, Stenzaly‐Achtert et al. [37] introduced aluminium chlorohydrate (ACH) into a deodorant formulation, this resulted in a slightly acidic deodorant‐antiperspirant product with a pH of 5. Although ACH is an active ingredient of antiperspirants, it also shows an antimicrobial property at low concentrations and has low pH (3.7–4.1) [38]. Nowadays, antiperspirant agents such as ACH and aluminium zirconium pentachlorohydrate are marketed as a deodorant‐antiperspirant.

The addition of fragrance helps in bringing down the unpleasant body odour. Linalool, hexylcinnamaldehyde, and 2‐(4‐tert‐butylbenzyl) propionaldehyde are commonly used fragrance compounds. Additionally, a study conducted by Kim et al. [34] revealed that most deodorants contained more than one type of fragrance compound and the concentration was in the range of 10^2^–10^4^ mg/kg.

### Testing methodology to evaluate the efficiency of deodorant products

**FIGURE 4:**
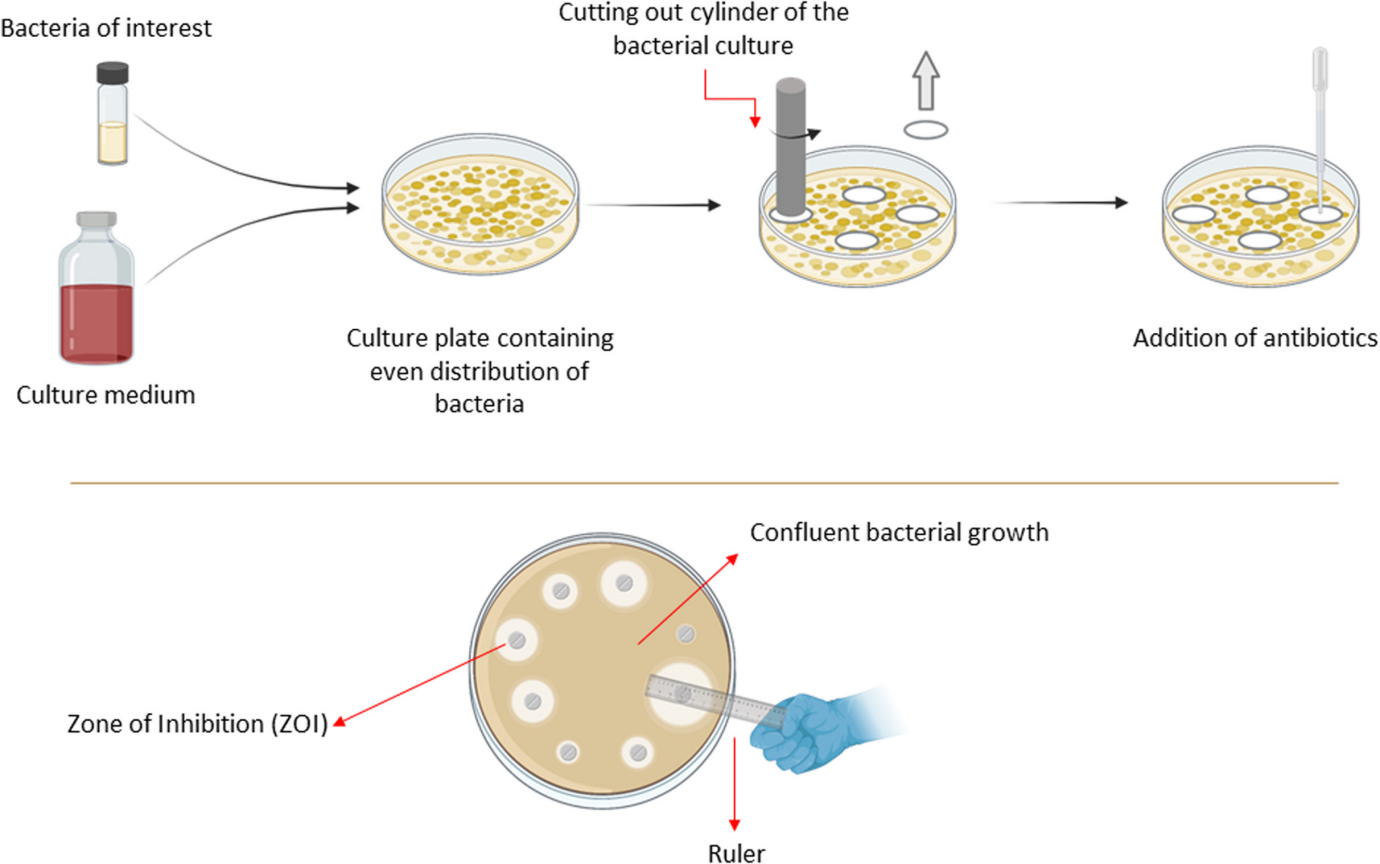
Schematic representation of the Kirby‐Bauer approach to quantify the Zone of Inhibition (ZOI), created with Biorender.com.

**FIGURE 5:**
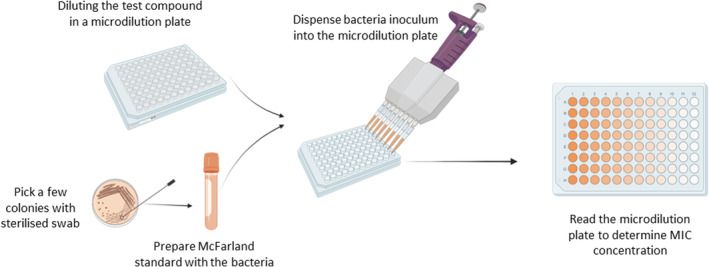
Schematic representation of the broth dilution assay in order to obtain the Minimum Inhibitory Concentration (MIC), created with Biorender.com.

As the mechanism of action of deodorants is to inhibit the growth of malodour‐forming bacteria, evaluation of the Zone of Inhibition (ZOI, illustrated in Figure 4) and the Minimum Inhibitory Concentration (MIC, illustrated in Figure 5) of the active ingredient are often an indicator of a deodorant's efficacy [39]. These two parameters are obtained by utilizing in vitro approaches. Kirby‐Bauer method is a qualitative approach used to acquire the ZOI [40]. The deodorant active ingredient is placed as an antibiotic disc onto the culture plate of malodour‐forming bacteria. Then the bacterial cell culture will incubate for 24 h or as long as needed, depending on the bacteria species [41]. The ZOI is measured by an empty area around the antibiotic disc (in the unit of millimetres, mm) where the bacteria have not grown enough to be visible. Depending on the size of the circular zone, antibiotics will be classified as resistant, susceptible, or intermediate based on the Clinical and Laboratory Standards Institute (CLSI) database.

MIC is described as the lowest concentration of the antibiotic that can prevent the visible growth of bacteria, expressed in a unit of μg/L or mg/L. To evaluate MIC, the broth dilution assay is used [42]. In summary, various concentrations of antibiotics are prepared in test tubes, followed by incubating those solutions with cultured bacteria. If bacteria grow, the solution will appear cloudy. The MIC value is identified at the concentration of antibiotics which produces a clear solution. Similar to the ZOI, this concentration will then be compared to the CLSI database, to determine whether the bacteria are resistant, susceptible, or intermediate to the antibiotic. However, it is important to note that these in vitro approaches have limitations. For example, the concentration of deodorant active ingredients remains constant throughout the Kirby‐Bauer analysis, whereas in real‐life application, it can be absorbed into the skin or evaporated. Therefore, this technique might not fully replicate real‐world conditions.

In addition to in vitro approaches, the efficiency of a deodorant product could also be measured quantitatively via using gas sensors. In 1996, Fujimoto et al. [43] introduced the concept of using a SnO_2_ semiconductor‐based gas sensor to measure the change of malodour gas concentrations after deodorants are being introduced into the test chamber. This approach allows the efficiency of the deodorant to be measured quantitatively at low cost and high accuracy. Although this study used active carbon and porous iron as their test substances, it has inspired many other researchers to use gas sensors for detecting the deodorizing property in the interest of personal care products [44, 45, 46].

Later, Caroprese et al. [47] published a study on utilizing the GC‐MS technique to evaluate the efficiency of deodorant products quantitatively. The major advantages of GC‐MS are that it is a general‐purpose analytical technique that is available in most laboratories and it is non‐invasive to the test subject involving in the study. Furthermore, GC‐MS additionally allows qualitative analysis, meaning that the chemical composition of eccrine and apocrine secretions can be identified. The results from this study showed a significant reduction (ranging from 26.6% to 77.0%) in the malodour acid content when deodorant cream was applied to the test subjects. However, the number of subjects in this study is limited (six people). It is critical to expanding the scale of this protocol, as the chemical composition and the concentration of malodour compounds can be significantly different from one individual to another.

## RESEARCH PROGRESS ON ANTIPERSPIRANT PRODUCTS AND COMPOSITION COMPONENTS

### Aluminium salts and their mechanism of action

Aluminium chloride in an aqueous solution was introduced in 1916 by Stillians [48] as an antiperspirant active and it is still considered to be one of the most effective antiperspirant ingredients. However, because of the acidic nature of aluminium chloride, it can cause skin irritation and clothes damaging which limited its use [49, 50]. Later in the 1940s, a less acidic aluminium chlorohydrate (ACH) active was introduced to the market [7]. The formulation consisted of salicylic acid, which reduced the incidence of skin irritation and also had good anti‐bacterial and anti‐fungal properties. Table 2 describes typical ingredients found in a commercially available antiperspirant formulation.

**TABLE 2:** Common ingredients in antiperspirant formulation, adapted from Benohanian 2001, Jungermann 1974, and Martini 2020 [5, 31, 56].

Role	Compound
Gel‐plug formation	Aluminium salts: aluminium chlorohydrate, aluminium sesquichlorohydrate, aluminium bromide, aluminium zirconium tetrachlorohydrate, and aluminium lactate [50]
Carrying vehicle	Ethanol, propylene glycol
Propellants (in the case of aerosol formulation)	Butane, isobutane, and propane
Fragrance	Limonene, linalool, geraniol, benzyl salicylate

The mechanism of action of antiperspirant is by the formation of gel plugs in sweat pores [51]. This prevents sweat from emerging onto the skin surface, keeps the axillary dry, and eliminates the food source for the bacteria. A gel plug is formed as a result of the interaction between aluminium salts and the biomolecules in the sweat solution. This interaction is first introduced as the “Gel Plug Theory” by Reller and Luedders [9]. Reller and Luedders observed an amorphous aluminium‐containing mass at the acrosyringium of the skin. They have hypothesized that the formation of this precipitate was likely due to the acidic aluminium salts being slowly neutralized as they travelled into the sweat duct and precipitated at the physiological pH.

**FIGURE 6:**
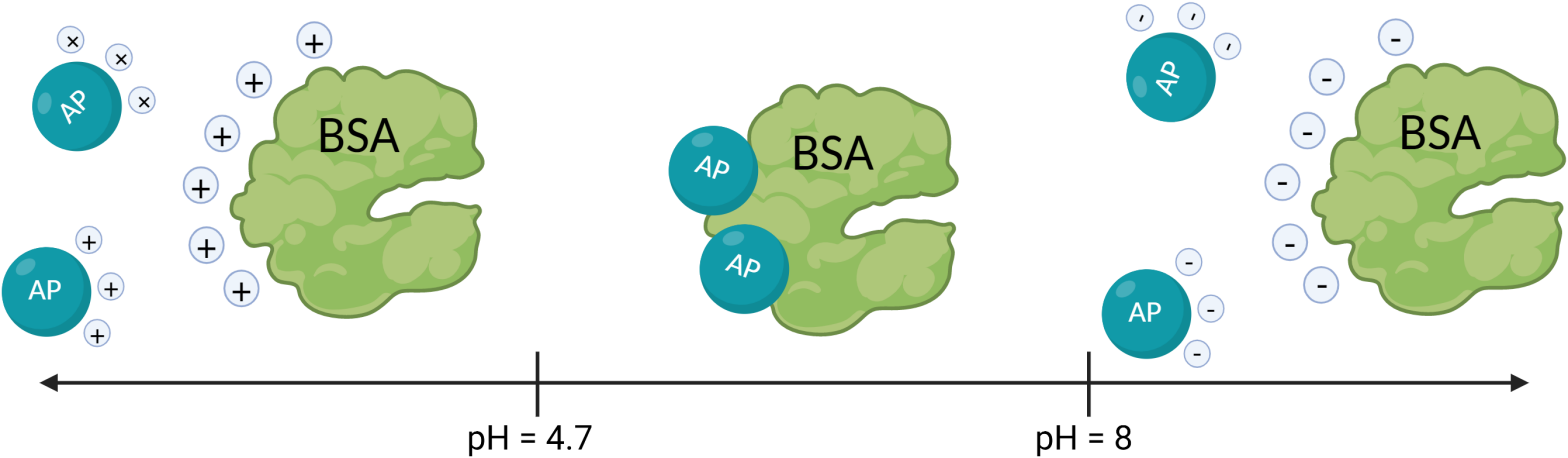
Schematic representation of the interaction between aluminium salts and BSA model protein, (adapted from Yuan et al. 2015).

Later in 2015, Yuan *et al* [52] have demonstrated this interaction using Bovine Serum Albumin (BSA) as a model protein in the sweat solution. According to the model, aluminium salts are hydrolysed into Al^3+^ ions upon contacting with BSA solution. The interaction between Al^3+^ ions and BSA is pH‐dependent and driven by electrostatic force as illustrated in Figure 6. At low pH (below 4), both species are cations and repulsed one another. However, at pH = 4.7, an isoelectric point of BSA protein is reached. This means the protein has an equal amount of negative and positive charges on its surface while Al^3+^ remains the same. Interaction of opposite charges species leads to the formation of water‐insoluble complexes.

### Testing methodologies for the evaluation of an antiperspirant's efficiency

For antiperspirants, their ability to reduce the amount of sweat is measured by a gravimetric approach involving human volunteers [53]. For instance, perspiration was collected before and after the exposure of antiperspirants using pre‐weight cotton pads which were placed under volunteers' axilla. Additionally, an aerosol antiperspirant formulation may be subjected to a fabric staining test. This is usually performed by observing the axilla region of t‐shirts worn by test subjects using a reflectometer.

Furthermore, a sensory assessment is conducted to evaluate the ability of an antiperspirant product in reducing the offensive body odour [54]. This assessment involves human volunteers and a panellist of trained sniffers. Firstly, the test subjects are required to undergo a wash phase, where they are given an unscented soap and a fresh t‐shirt to wear throughout the experiment duration [55]. It is crucial to avoid possible negative effects on the olfactory assessment. Therefore, the assessment is conducted odour‐neutral laboratory with defined ambient conditions and the test subjects are prohibited from using any other body odour treatment using the wash phase. After the wash phase is completed, a deodorant or antiperspirant product will be applied onto the test subject's axilla. Then the trained sniffers will sniff directly at the axilla, or a t‐shirt worn by the test subject after a certain period of time. Sniffers are trained to correlate their individual odour perception with an intensity calibration. According to the European Standard EN 13725:2003, 1‐butanol diluted in nitrogen gas is used as a reference odour substance. There are currently around 300 laboratories worldwide that use this method [56].

Even though the training protocol for the trained sniffers is established, the sensory assessments from two independent laboratories cannot be compared. This is because each laboratory can decide when will they going to carry out the sniffing testing, and where will be the site of assessment. For instance, Traupe et al. [54] carried out the assessment 24 and 48 h after application of the deodorant product. Then followed by sniffing directly at the axillary region of the test subject. Whereas Yoshioka et al. [55] performed the sniffing test at a t‐shirt worn by the participant immediately after the test subject performed sweat‐inducing activities such as exercise and performing stressful calculations.

**FIGURE 7:**
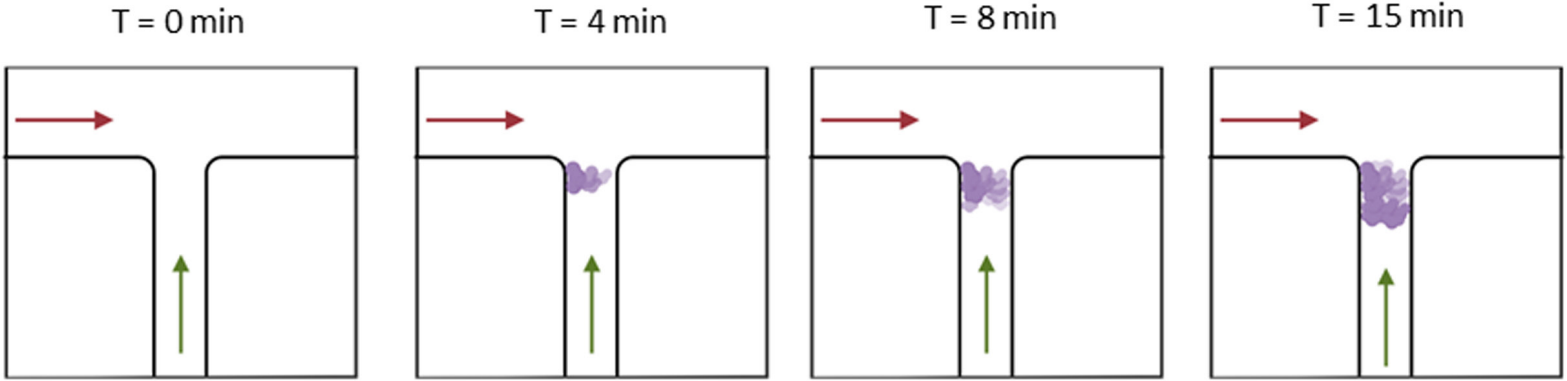
A schematic representation of the ACH gel‐plug formation observed in a microfluidic device with channel width of 50 μm. The horizontal arrow represented the flow direction of 15% ACH in aqueous solution and the vertical arrow represented the flow of sweat solution. Adapted from Bretagne et al. 2017 and created with Biorender.com [57].

In addition to in vitro and in vivo approaches conventionally used to evaluate the efficiency of deodorant and antiperspirant final formulations or commercial products, many researchers are working towards creating a platform to develop a better understanding of their mechanism of actions [57]. In 2017, Bretagne et al. [57] utilized a T‐junction microfluidic device to mimic a small channel nature of the human eccrine sweat ducts (illustrated in Figure 7). The objective of this experiment was to investigate the importance of proteins in the gel‐plug formation of antiperspirant actives. In this study, Bovine Serum Albumin (BSA) protein was selected as a model protein to represent the biomolecules found in sweat gland's secretions. The experiments were conducted in 3 different conditions: using natural sweat solution, using “artificial” sweat solution with BSA, and artificial sweat solution without BSA.

The authors have concluded that the presence of protein is crucial for the gel‐plug formation phenomenon of antiperspirant active agents. Furthermore, the mechanism of action can be categorized into 2 stages, the nucleation and the growth stages. The nucleation stage happened where the flow of antiperspirant agent met the sweat solution. The water‐insoluble gel plug attached to the wall of the microfluidic device and formed a tenuous membrane. Later in the growth stage, the membrane formed was collecting protein molecules from the sweat flow and expanding its size. The size of this membrane was growing until the channel is fully blocked.

According to the findings from this study, a microfluidic device was an excellent tool to observe interactions between sweat solution and ACH which leads to gel‐plug formation. Furthermore, the device used in this study has simplified the sweat duct geometry and the surrounding environment, making it suitable for finding new antiperspirant actives in the future, based on their interaction with sweat proteins. However, it is important to note that the experiment condition of the microfluidic device might not fully replicate real life conditions. In this study, the flow of both sweat and ACH were continuous throughout the duration of the experiment. Whereas in reality, an antiperspirant is applied only for a short period of time.

## ALTERNATIVE ACTIVE INGREDIENTS FOR BODY ODOUR REDUCTION TREATMENTS

Globally, there is a growing interest in using naturally derived ingredients such as plant extracts and essential oils in personal care products [7]. Nature‐derived compounds are often perceived as a safer and more environmentally friendly option than synthetic compounds by consumers. The current progress on developing novel and naturally derived alternative ingredients for both deodorants as well as antiperspirants is highlighted in this section.

### Naturally derived ingredients

#### Bacterial extracts

**FIGURE 8:**
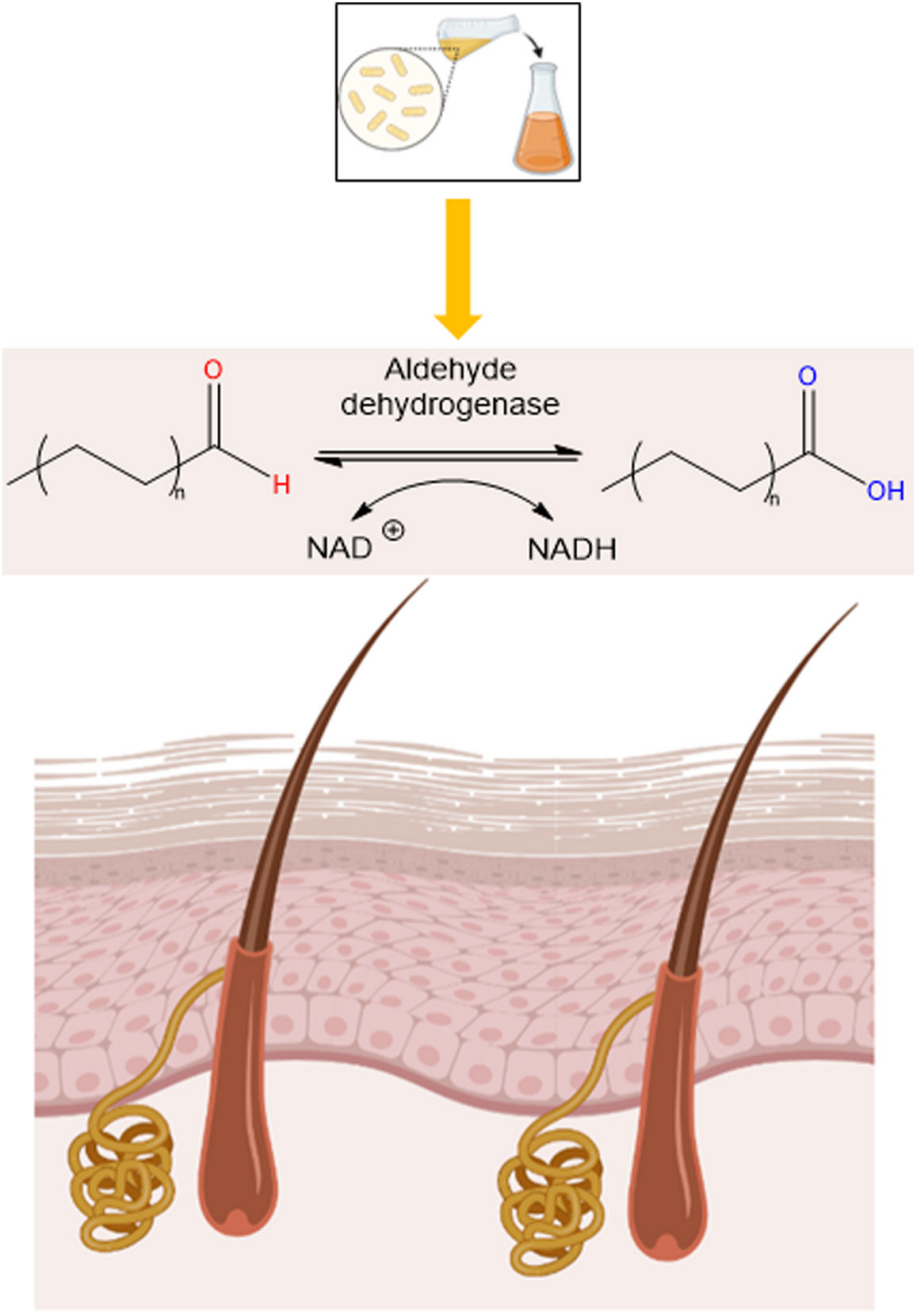
The mechanism of action of aldehyde dehydrogenase from AAB extracts (Created with BioRender.com, adapted from Yoshioka et al. [55]).

The use of acetic acid bacterial (AAB) extracts as a deodorizing agent was investigated by Yoshioka et al. 2018 [55]. AAB is a class of gram‐negative bacteria widely used in the food industries such as the production of vinegar and kombucha [58]. The cell membrane of AAB is comprised of two enzymes, alcohol dehydrogenase, and aldehyde dehydrogenase. The authors hypothesised that AAB extract can directly target the root cause of the unpleasant body odour, aldehyde [23], by converting aldehydes (R‐CHO) into carboxylic acids (R‐COOH) (mechanism of action shown in Figure 8).

In this study, the suspension solution of AAB extract was applied to the test subject's underarm. The concentration of malodourous aldehydes was measured using a thermal detector Gas chromatography/Mass spectrometry (GC‐MS) system and the sensory assessment was carried out by a group of trained sniffers. After application of AAB extract, the concentration of five aldehydes contributed to body odour (2‐nonenal, n‐hexanal, n‐heptanal, n‐octanal, and n‐nonanal) was much lower than the control group with DI water. The results obtained from this study showed that the AAB extract can significantly reduce the concentration of aldehyde compounds in body odour, but not completely eliminate them. In terms of the sensory assessment, the authors have pointed out that “a few” unpleasant odours were picked up from the panellists. However, it should be emphasized that the chemical composition of human body odour consists of other compounds in addition to aldehydes such as fatty acids and thiols. Nonetheless, the findings from this study have highlighted the potential use of AAB extract as a deodorizing agent against malodour aldehydes. Perhaps this agent may be employed in combination with other deodorizing agents in the future.

#### Plant extracts

In 2009, Dumas et al. [40] conducted a study to evaluate the antimicrobial activity of the supercritical hop extract obtained from the hop plant (Humulus lupulus L., Cannabaceae). In this study, the antibacterial activity of the hop extract was tested against *Corynebacterium xerosis* and *Staphylococcus epidermidis*, the key contributors in human body odour production. Furthermore, an in vivo sensory evaluation in 42 human volunteers was conducted to identify the deodorant activity of the hop/zinc ricinoleate deodorant stick.

The hop extract used in this study was obtained by the supercritical extraction technique using CO_2_ at 320 bar and 40°C. The HPLC analysis of the hop extract showed the presence of alpha and beta‐acids which exhibit an antimicrobial property via causing leakage at the bacterial cytoplasmic membrane and inhibiting their nutrient transport. However, the hop extract is only susceptible to gram‐positive bacteria, according to the in vitro bacterial culture conducted in this study. The average ZOI of hop extract was reported to be 9 mm at concentrations of 1.5 and 2 mg/mL against *S*. *epidermidis*, and 12 and 15 mm for *C*. *xerosis*, at the concentration of 1.5 and 2 mg/mL, respectively.

To formulate a deodorant stick, the hop extract was combined with zinc ricinoleate which has odour‐binding property. The author further indicated that zinc ricinoleate was added to help slow down the oxidative degradation of acid components in the hop extract. The results from the sensory evaluation showed that the mean malodour score has dropped from 6.28 ± 0.70 to 1.8 ± 0.71 after 8 h of application. The malodour score remained low after both 12 and 24 h of application at 1.82 ± 0.74 and 2.24 ± 0.77, respectively. Although the results from this study confirmed both antimicrobial and deodorizing activities of the hop extract, it is important to note that acid components in the hop extract are prone to oxidative degradation. Therefore, the shelf‐life of hop extract‐containing deodorant products is needed to be addressed in the future research.

The antimicrobial activity of *Terminalia* spp. plant's extract was studied by Cock et al. [59]. The plant samples were acquired from three different origins: Australia, India, and South Africa. Solvent extractions of the leaf, fruit, and bark of the plant samples were performed using 5 solvents with different polarities. In this study, the antimicrobial property of plant extracts is evaluated in comparison to vancomycin, a commercially available antibiotic. The authors reported that solvent extraction with high polarity solvents resulted in a higher yield of the plant extract. The methanolic leaf extracts showed excellent growth inhibition for various types of bacteria, especially *C*. *jeikeium* and *S*. *epidermidis*, which are the significant contributors to body malodour production. Furthermore, the methanolic leaf extract showed comparable inhibition recorded from the vancomycin control.

The authors have concluded that the antimicrobial activity of the plant extract is likely due to a high tannin content found in these plants (1.3%–6.7% relative abundance was measured using HPLC‐MS, reported by the author's earlier work in 2015) [60]. Although tannins can be an excellent alternative to commercial deodorant active ingredients, it is also possible for tannins to behave as an antiperspirant agent. Tannin is a polyphenolic compound that has the ability to bind and precipitate protein via non‐specific bonding such as hydrogen bonding and hydrophobic interaction which is similar to the gel‐plug formation mechanism of antiperspirants [61]. However, the in‐situ gelation property of tannin was not investigated in this study. It is noteworthy to mention that tannin can produce a yellow or brown tint when dissolving in water. This might be a concern when trying to formulate it and can affect the appearance of the product.

#### Essential oils

Essential oil is the term used to describe an aromatic volatile liquid obtained from plant materials via steam distillation [62, 63]. The components of essential oil are lipophilic and highly volatile secondary plant metabolites with a molecular weight lower than 300 Da, for example, terpenes, allyl, and isoallyl phenols. Applications of essential oils are diverse. They can be used in the cosmetics industry, alternative medicine, and agro‐alimentary uses due to their antimicrobial activity.

Satureja species is a well‐known aromatic plant of the family Lamiaceae, related to rosemary and thyme [39, 64]. A study conducted by Vagionas and colleagues reported the use of essential oils from three Satureja species as an antimicrobial agent [39]. The chemical composition analysed by GC‐MS indicated that the Satureja essential oil consisted of over eighty components. There are 6 major components in the essential oil: spathulenol (11.9%), α‐bisabolol oxide‐B (8.77%), terpinen‐4‐ol (7.12%), linalool (6.03%), bornyl acetate (4.75%), and β‐bourbonene (4.19%). Although the author's aim was to evaluate the antimicrobial property of the essential oil against bacteria found in food products, the essential showed excellent antimicrobial property against *Staphylococcus epidermidis* with MIC = 0.37–0.98 mg/mL, depending on the Satureja species.

*Salvia lanigera Poir* is another example of a plant in the family Lamiaceae whose essential oil shows antimicrobial activity towards gram‐positive bacteria. MIC = 50 ± 0.4 μg/mL and 12.5 μg/mL against *S*. *aureus* and *S*. *epidermidis*, respectively, of *Salvia lanigera essential oils* was reported by Tenore et al. [41]. These results could support the suggestion of using essential oils obtained from plants family Lamiaceae as an alternative to conventional antimicrobial agents towards gram‐positive bacteria.

*Carum copticum* is a medicinal plant whose seed is extensively used as food preservatives in India [65]. The essential oil extracted from *C*. *copticum* is rich in thymol, γ‐terpinene, and ρ‐cymene. The antimicrobial activity of these compounds has been highlighted by many researchers [66, 67]. In 2002, Singh et al. [66] reported the use of essential oil extracted *C*. *copticum* as an antimicrobial agent. The authors have concluded that *C*. *copticum* is effective against a broad range of bacteria, including *S*. *aureus* and Corynebacterium diphtheriae. In the case of *C*. *diphtheriae*, the antimicrobial activity of *C*. *copticum* essential oil was greater compared to the control group using ciprofloxacin (zone of inhibition, ZOI of 26 and 23 mm, for the essential oil and penicillin, respectively).

In 2015, Suzuki et al. [62] evaluated the potential usage of essential oil from *Origanum vulgare* Linnaeus as an antimicrobial agent against body odour‐producing bacteria. The essential oil sufficiently showed antimicrobial activity against *Micrococcus luteus*, *Proteus vulgaris*, *S*. *epidermidis*, and *Corynebacterium xerosis*. Additionally, the morphological changes in the strains treated with the essential oil were observed under Scanning Electron Microscope (SEM). The chemical profile of the essential oil was identified by High‐Resolution Gas Chromatography (HR‐GC). According to HR‐GC, the essential oil has a high content of γ‐terpinene, carvacrol, and terpinen‐4‐ol at 30.5%, 15.7%, and 13.0%, respectively. From the results obtained from both Singh et al. 2002 and Suzuki et al. 2015, it can be concluded that γ‐terpinene can be considered as a Corynebacterium growth inhibitor.

Recent research published by Moon et al. [44] reported the deodorant activity of essential oil extracted from *Asiasarum heterotropoides*. In this research, the essential oil was extracted using a system comprised of supercritical carbon dioxide fluid (Sc‐CO_2_) which was proven to be a more environmentally friendly approach compared to conventional organic solvent extractions and steam distillation. This is due to the use of non‐toxic, non‐flammable solvents, and at a lesser amount. Furthermore, the results from this study demonstrated that the supercritical fluid system yielded a greater amount of essential oil compared to solvent extraction.

The extracted essential oil demonstrated excellent deodorant activity with ammonia as representative odorous gas. During the first 10 min after the oil is introduced, over 80% of ammonia concentration was reduced and the concentration became nearly 0% after 30 min. Chemical compositions of the extracted oil were identified using GC‐FID and GC‐MS techniques. Over 27 volatile organic compounds were found, including methyleugenol which exhibits antimicrobial and antifungal properties. However, the antimicrobial of the extracted oil from this study was not reported.

### Synthetic ingredients

#### Anticholinergic agents

**FIGURE 9:**
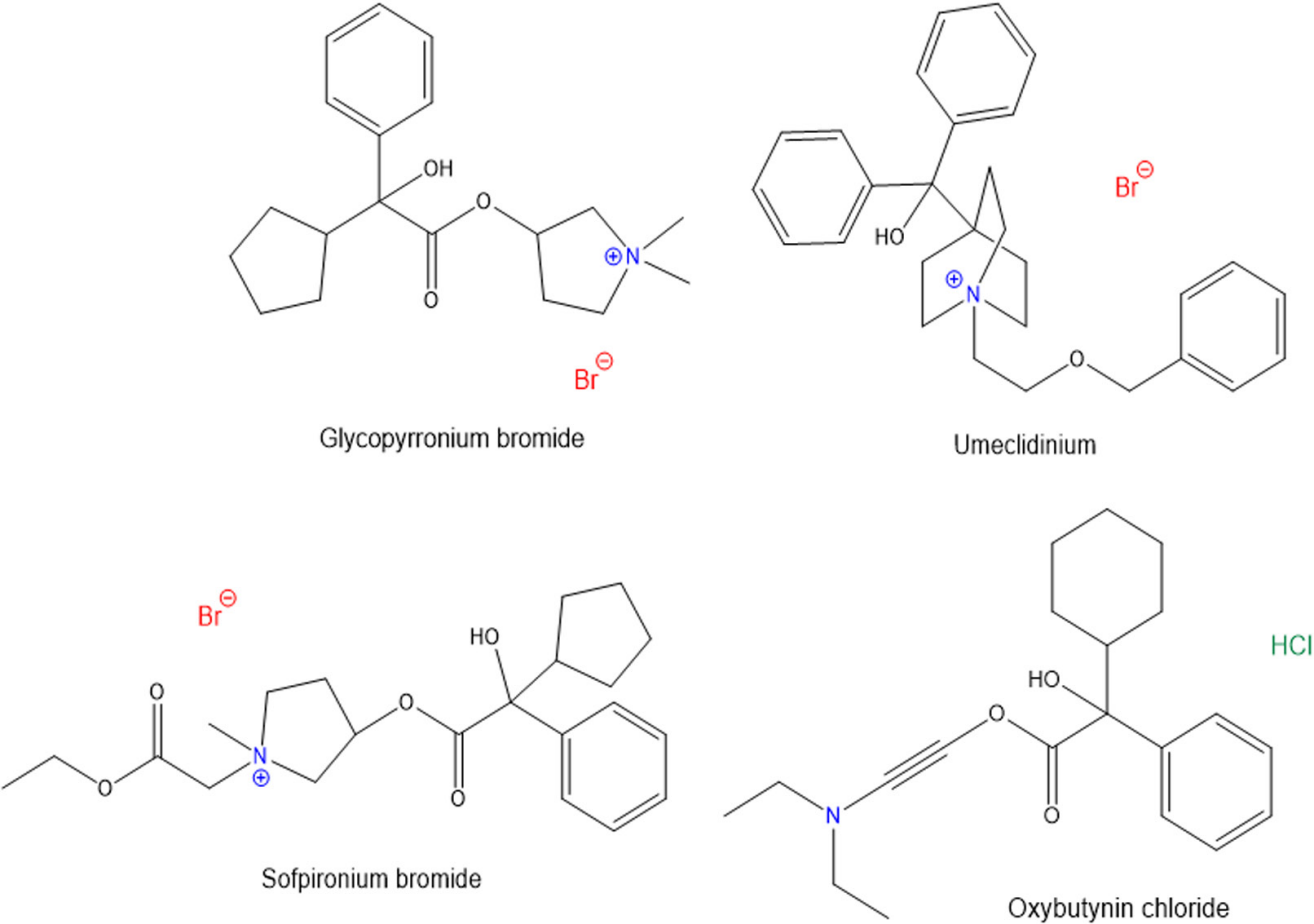
Chemical structures of common anticholinergic substances [68, 79, 80].

Anticholinergic is a term used to describe a class of substances that blocks the action of the neurotransmitter called acetylcholine (Ach) [68]. The mechanism of action of anticholinergics is to inhibit the binding of Ach to the muscarinic acetylcholine receptor (M Ach receptor) and bind themselves to the receptor instead. This receptor is responsible for the contraction of smooth muscles and increasing the amount of fluid secreted from sebaceous and sweat glands. For this reason, anticholinergic agents have gained interest over the recent years as an alternative treatment to an aluminium‐containing antiperspirant for hyperhidrosis patients. The majority of anticholinergic agents contain a highly polar quaternary ammonium group, this restricts their passage across human lipid membranes. The chemical structures of common anticholinergic compounds are shown in Figure 9.

#### Block copolymers

**FIGURE 10:**
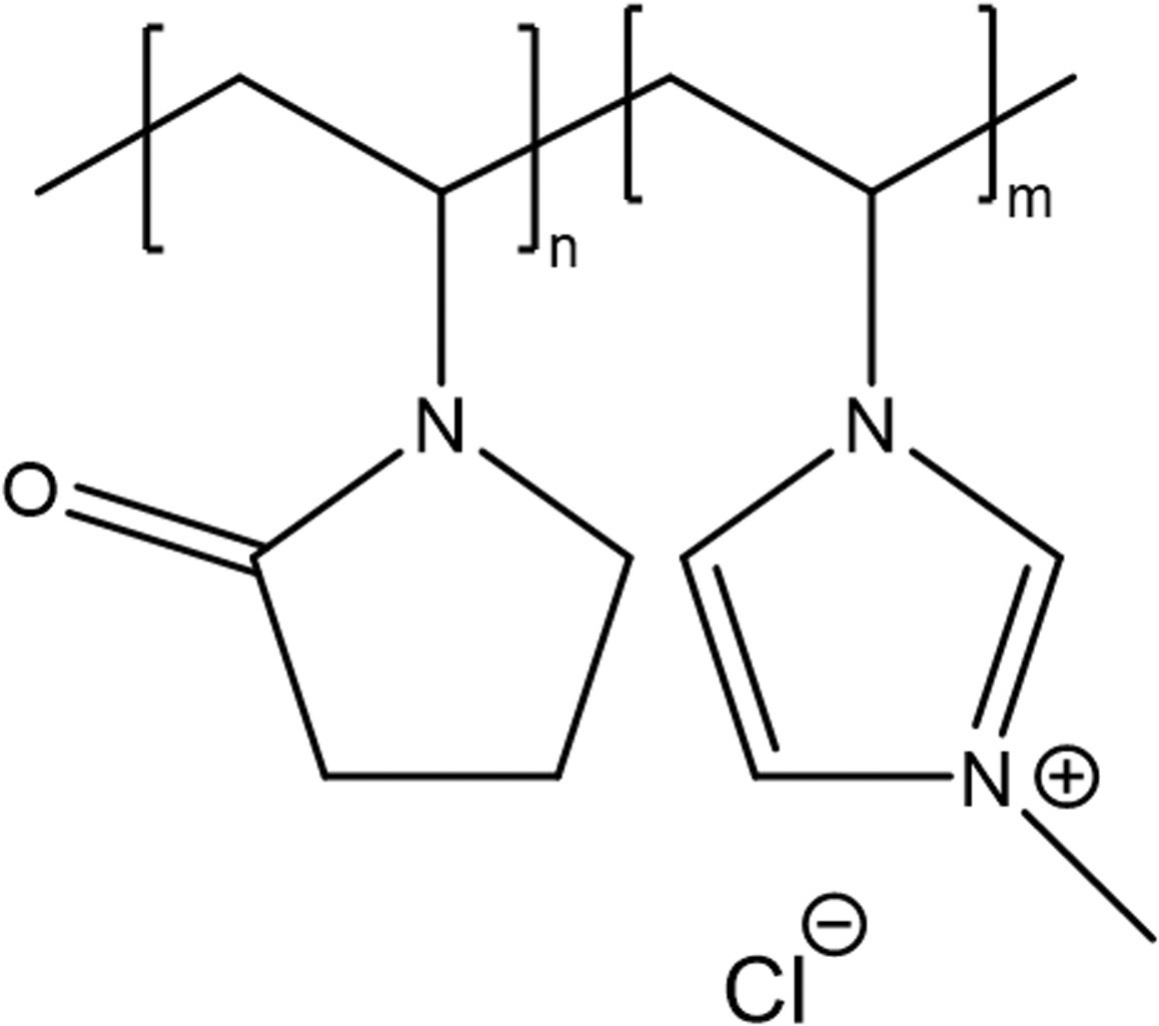
Polyquaternium‐16 (PQ‐16).

In 2017, Traupe et al. [54] studied the use of a synthetic cationic block copolymer as a deodorant active ingredient. In this study, the antimicrobial property, and the axilla odour intensity assessment of polyquaternium‐16 (PQ‐16) were investigated in vivo. PQ‐16 is a block copolymer of 1‐vinyl‐2‐pyrrolidone and 1‐vinyl‐3‐methylimidazolium chloride. The chemical structure is shown in Figure 10. This type of compound is already utilized in hair care products such as conditioners and shampoos. This is due to the presence of strong positive charges that can induce cytoplasmic membrane damage of bacterial and ionically bind to the hair. In addition, PQ‐16 has a high molecular weight, approximately 40 000–400 000 Da which is unlikely to penetrate to the skin (Robert et al. 2013) [69]. In this study, both antimicrobial property and axilla odour intensity assessment of the PQ‐16 deodorant were compared with a commercially available roll‐on deodorant and a perfumed microemulsion consisted of 10% ACH.

The authors have reported no adverse reaction or discomfort observed during the duration of this study and the roll‐on formulation consisted of PQ‐16 was well‐tolerated among the study population. After 1, 4, 8, 24, and 48 h of application, the bacterial count in the case of PQ‐16 roll‐on was reported to be significantly lower than the commercially available roll‐on. However, when comparing with the 10% ACH formulation the decrease in bacterial count was not significant. The axilla odour intensity assessment was evaluated by a panel of trained sniffers. The odour intensity score was given by the trained sniffer with a score ranging from 0 to 5 (0 means no odour detected and 5 means very strong axilla odour). It is observed that after 24 and 48 h of application, the trained sniffers rated the axilla odour intensity score of PQ‐16 roll‐on lower than the commercially available roll‐on but roughly the same with the 10% ACH formulation. Based on the bacterial count and axilla odour intensity score results, it can be concluded that the PQ‐16‐containing roll‐on formulation performed better than the commercially available roll‐on and provided comparable efficiency to the ACH‐containing formulation.

#### Silver nanoparticles (AgNPs)

Silver nanoparticles (AgNPs) are well known for their inhibitory and bactericidal effects. A study conducted by Guzman et al. [70] reported the ZOI and MIC of AgNPs against gram‐positive bacteria (*S*. *aureus*) and gram‐negative bacteria (*E*. *coli* and *P*. *aeruginosa*). It is observed that their antibacterial property is influenced by particle size. Smaller particle size offers a higher surface area available for the interaction to occur than larger particle. Hence, higher antimicrobial activity was observed. The mechanism of action of AgNPs was purposed by Feng et al. [71] and can be summarized into two steps. Firstly, the nanoparticle is attached to the surface of a bacterial cell wall, followed by releasing Ag^+^ ions. Secondly, the released Ag^+^ enters the cell, interacting with bacterial DNA and proteins. Ag^+^ turns the bacterial DNA into its condensed form which then leads to inhibition of DNA replication. In addition, Ag+ interacts with the thiol group in proteins, inducing the inactivation of the proteins.

Bellarin et al. [72] reported a simplistic approach to synthesis a hybrid material containing ZnAl layered double hydroxides (ZnAl LDHs) and silver nanoparticles (AgNPs). LDHs are anionic clay compound which is abundant in nature and easily synthesis in the laboratory. It is employed in various applications such as using as an adsorbent, catalytic support, and ion exchangers. In addition, it is well‐known for its high adsorption capacity. For AgNPs, it has gained attention in biomedical applications due to its antimicrobial activity. The authors aimed to combine both materials together to create a hybrid material that is bio‐compatible, high adsorption capacity, and antimicrobial activity.

The adsorption of AgNPs onto ZnAl LDHs substrate was confirmed by X‐ray diffractometer (XRD), Fourier‐transform infrared spectroscopy (FT‐IR), and SEM. The deodorant activity was evaluated with a mixture of fatty acids to replicate the malodour formed as a result of perspiration. The authors showed that the deodorant activity of this hybrid material was higher than zinc ricinoleate. The antimicrobial activity of ZnAl‐ AgNPs was observed with *Escherichia coli* via using in vitro turbidimetric analysis. However, the authors did not report the antimicrobial activity for bacteria responsible for human body odour.

#### Deodorizing fabrics

Thus far, researchers are focusing on finding an alternative deodorizing agent that can be incorporated into topical deodorant or antiperspirant products. However, the application of topical products is not the only approach to target body odour. Several research groups have investigated the use of antimicrobial or deodorizing fabric to overcome this problem. In 2011, Stay Fresh® antimicrobial fabric treatment developed by Quick‐Med Technologies Inc. received the U.S. Environmental Protection Agency (EPA) registration for its usage in textile applications such as clothing, interior furniture, and carpet [73]. Hydrogen peroxide is bonded onto the fabric and act as an active ingredient to provide gentle disinfectant property and bleaching action to the fabric. However, this technology is still under development and awaits its approval from the FDA.

Research published by Zhang et al. [45] demonstrated the deodorizing property of photocatalyst (nanoscale‐TiO_2_) containing textiles. Nanoscale‐TiO_2_ can absorb oxygen and capture free electron under the presence of light, generating O^2−^ ions which oxidize and decompose various organic gases. In this study, a series of polyester and bamboo fibre blended textile with different compositions of nanoscale‐TiO_2_ (0%–100%) was prepared. The deodorizing property of those textile was evaluated using ammonia (NH_3_) as a representative odour gas. It was observed that after 12 h, all textile samples can reduce the concentration of ammonia gas. As expected, the deodorizing rate was increasing with increasing in TiO_2_ composition. Although all the textile samples in this study showed some deodorizing properties against NH_3_ gas, further investigation with malodour aldehydes, carboxylic, and thiol gases is necessary.

Later in 2018, the deodorizing property of phthalocyanine complex nanofibers was investigated by Lee et al. [46]. Phthalocyanine (Pc) is a conjugated aromatic compound frequently used as a dye molecule. It has excellent thermal and chemical stability, low‐cost advantage, and non‐toxicity. Moreover, it is also been known as an antioxidizing agent against malodour gases such as hydrogen sulphate and thiols. Through cyclic oxidation and reduction reaction, phthalocyanine can act as a catalyst to convert foul‐smelling odour compounds into odourless molecules without being consumed. Nanofibers in this study were fabricated by incorporating 4 wt% of Cu‐coordinated Pc into a polymeric solution of poly(vinyl alcohol), (PVA) and silk. The nanofibers were prepared by electrospinning technique, resulting in a fibre diameter of 403 ± 80 nm (Pc‐PVA) and 534 ± 74 nm (Pc‐Silk).

The deodorant activity of phthalocyanine‐PVA and phthalocyanine‐silk nanofibers was investigated by measuring the reduction in concentrations of methanethiol gas, which is a model odorant in this study. The reduction in malodour gas concentration was reported to be 15% and 40% for pure PVA and silk nanofibers, respectively. The authors suggested that this is likely due to the large surface area of both materials. Additionally, incorporating phthalocyanine into the nanofiber, the concentration of methanethiol gas dropped significantly (25% for Pc‐PVA and 50% for Pc‐Silk).

Based on the findings of this investigation, it is reasonable to conclude that nanofibers have the potential to reduce the concentration of malodour gas through physical absorption. Additionally, incorporating Pc molecules can exhibit excellent deodorizing properties. Although methanethiol is one of the chemical components found in human body odour, they are primarily identified at the plantar, rather than the axilla region. Further investigations with malodour compounds found at the axilla (such as HMHA and 3M3SHH) are needed in order to evaluate the potential usage of phthalocyanine nanofibers as deodorizing fabric.

#### Hygroscopic materials

A recent study conducted by Lolla et al. [74] illustrated the use of hygroscopic material (material that can absorb water from its surroundings) to induce evaporation and self‐clogging phenomenon of eccrine sweat glands secretion. Eccrine sweat glands secreted watery fluid that is rich in mineral content including NaCl, KCl, and bicarbonate. Therefore, the authors of this study have hypothesised that the mineral content of sweat could potentially lead to self‐clogging phenomena when the water component is evaporated. In this study, an artificial sweat duct was created and observed under a microscope. A glass tube with two openings with a diameter of 50 μm was used to mimic the microchannel nature of eccrine sweat glands. One end of the glass tube was connected to a syringe which introduced an artificial sweat solution to the tube at a controlled flow rate. Another end of the tube is either opened to air, exposed to a dry plastic cube made of PDMS, and exposed to propylene glycol infused PDMS which is the hygroscopic material in this study.

**FIGURE 11:**
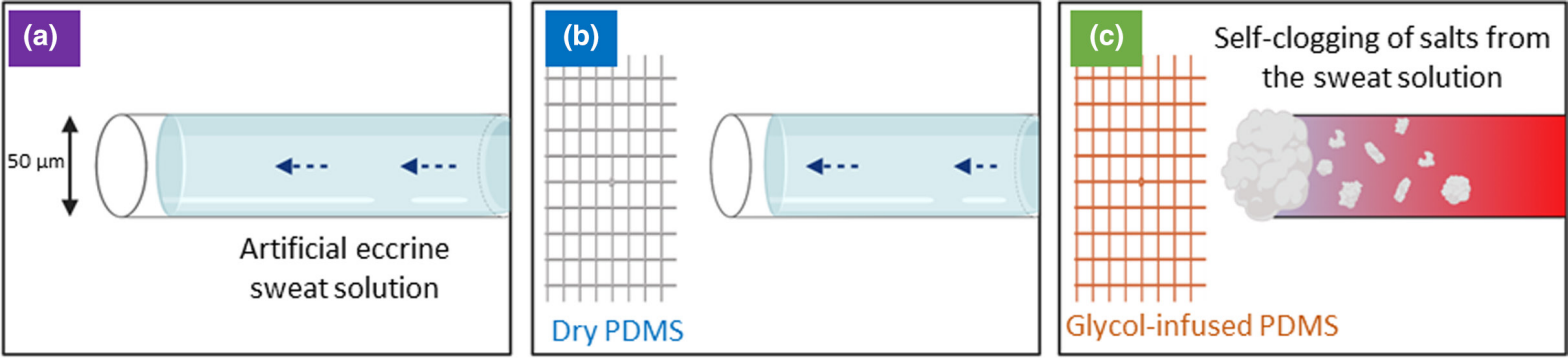
Schematic representations and microscopic images obtained during the experiment when (a) the end of the glass tube was freely opened to air, (b) a dry PDMS cube was placed closed to the end of the tube, and (c) a propylene glycol‐infused PDMS cube was placed. Adapted from Reference [75].

According to Figure 11, the pressurized sweat fluid can flow pass through in both opened to air and in the presence of PDMS cube cases. In contrast, the self‐clogging phenomenon was observed in the presence of propylene glycol. The plug formed was reported to obstruct the sweat flow for over an hour. However, it was also reported that sweat flow can be stopped (but no gel‐like material being formed) without the presence of propylene glycol when the back pressure is decreased below 1.725 kPa due to the capillary resistance of a small diameter glass tube.

The findings of this research indicate that self‐clogging of eccrine sweat fluid can result in a gel‐plug similar to that created by the use of traditional aluminium salt antiperspirants. Additionally, propylene glycol is non‐toxic, readily available, and has low‐cost advantages. Nevertheless, it is important to note that further in vivo investigations are needed, not only to evaluate the effectiveness of the hygroscopic materials, but also to determine the ease and the comfort level of using them as antiperspirant sheet.

## CHALLENGES IN DEVELOPING ALTERNATIVE DEODORANT AND ANTIPERSPIRANT ACTIVE INGREDIENTS

The vast majority of the research on developing an alternative body odour reduction treatment are focusing on deodorant's mechanism of action rather than antiperspirants. Although plant extracts and essential oils can both take action as antimicrobial agents and provide a pleasing smell, those ingredients might not be suitable for individuals with strong body odour or hyperhidrosis patients. Moreover, the use of plant extracts and essential oils in deodorant or antiperspirant products might be limited by their country/region of origin and their availability. While the novel deodorizing agents presented in this review article have shown excellent antimicrobial activity towards human natural microflora, it is crucial for those ingredients to be compared to commercially available product and be subjected to a sensory assessment as the one of the aims of deodorants and antiperspirants products is to reduce the axilla odour intensity. In addition to antimicrobial activity and sensory assessments, patch testing is also highly encouraged. It should be noted that a large‐scale production of deodorant with plant extracts or essential oils can be a challenging and costly process. Extraction processes often require a large volume of solvent and long extraction time. Lastly, the general concerns of most antimicrobials are that they can lead to resistant bacterial strains being developed, making the product less effective over time.

## CHALLENGES IN DEVELOPING TESTING METHODS FOR ANTIPERSPIRANT PRODUCTS

In terms of antiperspirant testing methods, there should be a guideline to conduct an in vivo sensory assessment to make the results from different laboratories comparable to each other. For instance, a set temperature of the hot room laboratory, a defined activity for the test subject to conduct in order to induce sweat, and the time period where the trained sniffer sniffing at the test subject's axilla. Additionally, the majority of existing testing methods for antiperspirant actives are relying on their ability to reduce the foul body odour or the amount of perspiration at the axilla. However, a platform to develop a more thorough understanding and to observe the aluminium salt's mechanism of action is not widely reported in the literature. Therefore, developing a device similar to the microfluidic device reported by Bretagne et al. [57] might be helpful as a screening device for finding a new antiperspirant agent.

Nevertheless, there are certain aspects of the microfluidic device to be improved in order to better mimic human sweat glands at the axilla. The first aspect is the operating condition of the device, as the human's axilla is a warm and moist body part [75]. Secondly, the channel of the microfluidic device is made up of synthetic materials, unlike the human sweat duct where epithelial cells and various proteins are present [76]. Coating the inner channel with proteins or addition of components found in the human sweat duct could potentially affect the in‐situ gelation phenomenon. Lastly, a human sweat duct has an irregular shape, and the diameter of the duct might be inconsistent [77]. Therefore, it is crucial to keep these features in mind while designing the testing device. Further study on developing a rapid screening method is necessary and incorporating biological elements into the method is highly encouraged.

To our knowledge, there have been no reported in‐vivo studies on the gel plug formation of antiperspirant actives. Nor have been reported studies on the dose–response kinetics of gel plug formation and eventual removal as affected by formulation property and sweat flow conditions. The efficacy and hence the formulation design of deodorants and antiperspirants products is mostly via empirical hot room studies. Many fundamental questions remain unanswered. For instance, what concentration of aluminium salts is delivered to sweat pores and what is the minimal concentration required in sweat pores to form gel plug? How is the eventual removal of gel plug affected by sweat flow and other formulation factors? Can other gelation technologies be exploited to deliver antiperspirant benefits and how?

## CONCLUSION

In modern society, human body odour may perceive as a sign of poor hygiene and affect an individual's self‐confidence level. Topical deodorants and antiperspirants are common approaches to target foul‐smelling body odour. However, the success of those products is relying on active ingredients that have been introduced more than 50 years ago. In this article, the investigation of the use of bacterial extracts, plant extracts, essential oils, and synthetic compounds as alternatives to conventional deodorant and antiperspirant active ingredients is summarized. The alternatives present in this article may have the potential to be incorporated into a deodorant formulation in the future but further investigation, especially comparing these alternatives with existing formulations is required.

## SUMMARY

Sweating undoubtedly has a critical role in regulating the body core temperature. However, sweating also results in body odour which may perceive as a sign of poor hygiene and affect an individual's self‐confidence level. To overcome the offensive odour, topical deodorants and antiperspirants are used. Although both products are considered to be the key sector in the personal care product industry, there is limited literature available on this topic. The current progress on deodorant and antiperspirant research is summarized in this review article.

Over the recent years, consumers have shown a growing interested in purchasing personal care products with naturally derived ingredients. In this review article, a new class of deodorant active ingredients, including essential oils, deodorizing fabric, plant, and bacterial extracts are present. Initially, the efficiency of deodorant products is identified by in vitro approaches to obtain ZOI or MIC. However, for the recent studies, a gas chamber is utilized for this purpose. This involves an analytical procedure that is readily available in most research laboratories such as gas chromatography to identify the reduction in malodour gas concentration after a deodorant product is introduced. Additionally, gas chromatography allows quantitative analysis. Therefore, the chemical composition of human body odour can be identified.

For antiperspirant products, their mechanism of action is based on the gel plug phenomenon of aluminium salts. The use of aluminium salts has been introduced since 1916, since then, the formulation has developed to be less acid, less fabric staining, and less irritated to the skin. To our knowledge, there is a limited amount of research on finding alternative antiperspirant actives. This could potentially be due to the lack of appropriate screening methodology for such ingredients. Most methodologies used to identify antiperspirant's efficiency are relying on human volunteer study which can be time consuming and costly process. There are several attempts to create a quick screening platform for new antiperspirant actives such as the turbidity measurement (Yuan et al. 2015) and a microfluidic device (Bretagne et al. 2017). Nevertheless, the biological components and complex structure of human sweat glands are still missing in these platforms.

## References

[1] Low PA . Chapter 51 – sweating. Primer on the autonomic nervous system. 3rd ed. Amsterdam: Elsevier; 2012. p. 249–51.

[2] Xhauflaire‐Uhoda E , Mayeux G , Quatresooz P , Scheen A , Piérard GE . Facing up to the imperceptible perspiration. Modulatory influences by diabetic neuropathy, physical exercise and antiperspirant. Skin Res Technol. 2011;17(4):487–93. 21438925 10.1111/j.1600-0846.2011.00523.x

[3] Havlicek J , Lenochova P . The effect of meat consumption on body odor attractiveness. Chem Senses. 2006;31(8):747–52. 16891352 10.1093/chemse/bjl017

[4] Kanlayavattanakul M , Lourith N . Body malodours and their topical treatment agents. Int J Cosmet Sci. 2011;33(4):298–311. 21401651 10.1111/j.1468-2494.2011.00649.x

[5] Benohanian A . Antiperspirants and deodorants. Clin Dermatol. 2001;19(4):398–405. 11535380 10.1016/s0738-081x(01)00192-4

[6] Laden K . Antiperspirants and deodorants: history of major HBA market. Antiperspirants and deodorants. 2nd ed. New York: Marcel Dekker; 1999. p. 1–17.

[7] de Oliveira ECV , Salvador DS , Holsback V , Shultz JD , Michniak‐Kohn BB , Leonardi GR . Deodorants and antiperspirants: identification of new strategies and perspectives to prevent and control malodor and sweat of the body. Int J Dermatol. 2021;60(5):613–9. 33644863 10.1111/ijd.15418

[8] Lai‐Cheong JE , McGrath JA . Structure and function of skin, hair and nails. Amsterdam, Netherlands: Elsevier; 2021.

[9] Reller HH , Luedders WL . Pharmacologic and toxicologic effects of topically applied agents on the eccrine sweat glands. Dermatotoxicology and pharmacology. New York: John Wiley and Sons; 1977. p. 1–54.

[10] Robinson JR . o‐t‐c deodorants and antiperspirants. J Am Pharm Assoc. 1967;7(2):75–93. 6045091 10.1016/s0003-0465(16)30107-0

[11] Schiefferdecker P . Über morphologische Sekretionserscheinungen in den ekkrinen Hautdrüsen des Menschen. Archives of Dermatological Research. 1921;132:130–2.

[12] Sato K , Kang WH , Saga K , Sato KT . Biology of sweat glands and their disorders. I. Normal sweat gland. J Am Acad Dermatol. 1989;20(4):537–63. 2654204 10.1016/s0190-9622(89)70063-3

[13] Schittek B , Hipfel R , Sauer B , Bauer J , Kalbacher H , Stevanovic S , et al. Dermcidin: a novel human antibiotic peptide secreted by sweat glands. Nat Immunol. 2002;2(12):1133–7. 10.1038/ni732 11694882

[14] Bovell DL . The evolution of eccrine sweat gland research towards developing a model for human sweat gland function. Exp Dermatol. 2018;27(5):544–50. 29626846 10.1111/exd.13556

[15] Wilke K , Martin A , Terstegen L , Biel SS . A short history of sweat gland biology. Int J Cosmet Sci. 2007;29(3):169–79. 18489347 10.1111/j.1467-2494.2007.00387.x

[16] Labors JN , Preti G , Hoelzle E , Leyden J , Kligman A . Steroid analysis of human apocrine secretion. Steroids. 1979;34(3):249–58. 158859 10.1016/0039-128x(79)90077-1

[17] Sato K , Leidal R , Sato F . Morphology and development of an apoeccrine sweat gland in human axillae. Am J Physiol Regul Integr Comp Physiol. 1987;252(1):R166–80. 10.1152/ajpregu.1987.252.1.R166 3812728

[18] Cheung JS , Solomon BA . Disorders of sweat glands: hyperhidrosis: unapproved treatments. Clin Dermatol. 2002;20(6):638–42. 12490357 10.1016/s0738-081x(02)00284-5

[19] Walder D , Penneys NS . Antiperspirants and deodorizers. Clin Dermatol. 1988;6(3):37–9. 3071402 10.1016/0738-081x(88)90030-2

[20] Callewaert C , Kerckhof F‐M , Granitsiotis MS , Van Gele M , Van de Wiele T , Boon N . Characterization of staphylococcus and Corynebacterium clusters in the human axillary region. PLoS One. 2013;8(8):e70538. 23950955 10.1371/journal.pone.0070538 PMC3741381

[21] Callewaert C , Hutapea P , Van de Wiele T , Boon N . Deodorants and antiperspirants affect the axillary bacterial community. Arch Dermatol Res. 2014;306(8):701–10. 25077920 10.1007/s00403-014-1487-1

[22] Amoore JE , Venstrom D , Davis AR . Measurement of specific anosmia. Percept Mot Skills. 1968;26(1):143–64. 5642519 10.2466/pms.1968.26.1.143

[23] Jha SK . Characterization of human body odor and identification of aldehydes using chemical sensor. Rev Anal Chem. 2017;36(2):1–16.

[24] Haze S , Gozu Y , Nakamura S , Kohno Y , Sawano K , Ohta H , et al. 2‐Nonenal newly found in human body odor tends to increase with aging. J Invest Dermatol. 2001;116(4):520–4. 11286617 10.1046/j.0022-202x.2001.01287.x

[25] Daniel B , Cox DS , Ashford D , James AG , Thomas GH . Identification of axillary *Staphylococcus* sp. involved in the production of the malodorous thioalcohol 3‐methyl‐3‐sufanylhexan‐1‐ol. FEMS Microbiol Lett. 2015;362(16):1–10. 10.1093/femsle/fnv111 26163522

[26] Starkenmann C , Niclass Y , Troccaz M , Clark AJ . Identification of the precursor of (S)‐3‐Methyl‐3‐sulfanylhexan‐1‐ol, the Sulfury malodour of human axilla sweat. Chem Biodivers. 2005;2(6):705–16. 17192014 10.1002/cbdv.200590048

[27] Natsch A , Schmid J , Flachsmann F . Identification of odoriferous Sulfanylalkanols in human axilla secretions and their formation through cleavage of cysteine precursors by a C‐S lyase isolated from axilla bacteria. Chem Biodivers. 2004;1(7):1058–72. 17191898 10.1002/cbdv.200490079

[28] Noe F , Polster J , Geithe C , Kotthoff M , Schieberle P , Krautwurst D . OR2M3: a highly specific and narrowly tuned human odorant receptor for the sensitive detection of onion key food odorant 3‐Mercapto‐2‐methylpentan‐1‐ol. Chem Senses. 2017;42(3):195–210. 27916748 10.1093/chemse/bjw118

[29] Stewart JCM . Tomatoes cause under‐arm odour. Med Hypotheses. 2014;82(5):518–21. 24576684 10.1016/j.mehy.2014.02.001

[30] Harker M , Carvell A‐M , Marti VPJ , Riazanskaia S , Kelso H , Taylor D , et al. Functional characterisation of a SNP in the ABCC11 allele—effects on axillary skin metabolism, odour generation and associated behaviours. J Dermatol Sci. 2014;73(1):23–30. 24076068 10.1016/j.jdermsci.2013.08.016

[31] Martini MC . Déodorants et antitranspirants. Ann Dermatol Venereol. 2020;147(5):387–95. 32248967 10.1016/j.annder.2020.01.003

[32] Cox AR . Efficacy of the antimicrobial agent triclosan in topical. J Soc Cosmet Chem. 1987;38:223–31.

[33] Lamb JH . Sodium bicarbonate: an excellent deodorant. J Invest Dermatol. 1946;7(3):131–3.

[34] Kim J‐H , Kim T , Yoon H , Jo A , Lee D , Kim P , et al. Health risk assessment of dermal and inhalation exposure to deodorants in Korea. Sci Total Environ. 2018;625:1369–79. 29996434 10.1016/j.scitotenv.2017.12.282

[35] Lukacs A , Korting HC , Braun‐Falco O , Stanzl K . Efficacy of a deodorant and its components: triethylcitrate and perfume. J Soc Cosmet Chem. 1991;42:159–66.

[36] Lukacs A , Korting HC , Lemke O , Ruckdeschel G , Ehret W , Braun‐Falco O . The influence of the pH‐value on the growth of Brevibacterium epidermidis in continuous culture. Acta Derm Venereol. 1995;75(4):280–2. 8578948 10.2340/0001555575280282

[37] Stenzaly‐Achtert S , Schölermann A , Schreiber J , Diec KH , Rippke F , Bielfeldt S . Axillary pH and influence of deodorants. Skin Res Technol. 2000;6:87–91. 11428948 10.1034/j.1600-0846.2000.006002087.x

[38] Ermenlieva N , Georgieva E , Milev M . Antibacterial and antifungal activity of antiperspirant COSMETIC products. J IMAB‐ Ann Proc (Scientic Papers). 2020;26(4):3374–7.

[39] Vagionas K , Graikou K , Ngassapa O , Runyoro D , Chinou I . Composition and antimicrobial activity of the essential oils of three Satureja species growing in Tanzania. Food Chem. 2007;103(2):319–24.

[40] Dumas ER , Michaud AE , Bergeron C , Lafrance JL , Mortillo S , Gafner S . Deodorant effects of a supercritical hops extract: antibacterial activity against *Corynebacterium xerosis* and *Staphylococcus epidermidis* and efficacy testing of a hops/zinc ricinoleate stick in humans through the sensory evaluation of axillary deodorancy. J Cosmet Dermatol. 2009;8:197–204. 19735518 10.1111/j.1473-2165.2009.00449.x

[41] Tenore GC , Ciampaglia R , Arnold NA , Piozzi F , Napolitano F , Rigano D , et al. Antimicrobial and antioxidant properties of the essential oil of *Salvia lanigera* from Cyprus. Food Chem Toxicol. 2011;49(1):238–43. 20977923 10.1016/j.fct.2010.10.022

[42] Ackerman BH , Dello Buono FA . In vitro testing of antibiotics. Pharmacotherapy. 1996;16(2):201–21. 8820464

[43] Fujimoto C , Hayakawa Y , Ono A . Evaluation of the efficiency of deodorants by semiconductor gas sensors. Sens Actuators B. 1996;32:191–4.

[44] Moon JN , Getachew AT , Haque AST , Saravana PS , Cho YJ , Nkurunziza D , et al. Physicochemical characterization and deodorant activity of essential oil recovered from Asiasarum heterotropoides using supercritical carbon dioxide and organic solvents. J Ind Eng Chem. 2019;69:217–24.

[45] Zhang H , Ge C , Zhu C , Li Y , Tian W , Cheng D , et al. Deodorizing properties of photocatalyst textiles and its effect analysis. Phys Procedia. 2012;25:240–4.

[46] Lee H , Jatoi AW , Kyohei Y , Kim K‐O , Song KH , Lee J , et al. Deodorant activity of phthalocyanine complex nanofiber. Text Res J. 2018;88(6):630–5.

[47] Caroprese A , Gabbanini S , Beltramini C , Lucchi E , Valgimigli L . HS‐SPME‐GC‐MS analysis of body odor to test the efficacy of foot deodorant formulations. Skin Res Technol. 2009;15(4):503–10. 19832965 10.1111/j.1600-0846.2009.00399.x

[48] Stillians AW . The control of localized hyperhidrosis. JAMA. 1916;LXVII(27):2015–6.

[49] Grote IW , Holbert JM , Cross PW . Dichloro aluminum aminoacetate, a new anti‐perspirant and deodorant. J Am Pharm Assoc. 1949;38(11):593–4. 10.1002/jps.3030381107 15392531

[50] Karsai S , Weiß C , Lütgerath C , Ott I , Faulhaber J . Comparison of novel aluminium lactate versus aluminium chloride‐based antiperspirant in excessive axillary perspiration: first prospective cohort study. Dermatol Ther. 2021;34(4):e15020. 34085372 10.1111/dth.15020

[51] Holzle E , Kligman AM . Mechanism of antiperspirant action of aluminum salts. J Soc Cosmet Chem. 1979;30:279–95.

[52] Yuan S , Vaughn J , Pappas I , Fitzgerald M , Masters JG , Pan L . Optimal aluminum/zirconium: protein interactions for predicting antiperspirant efficacy using zeta potential measurements. J Cosmet Sci. 2015;66(2):95–111. 26454974

[53] Maxeiner B , Ennen J , Rützel‐Grünberg S , Traupe B , Wittern KP , Schmucker R , et al. Design and application of a screening and training. Int J Cosmet Sci. 2009;31:193–9. 19563587 10.1111/j.1468-2494.2009.00494.x

[54] Traupe B , Fölster H , Max H , Schulz J . Effective axillary malodour reduction by polyquaternium16‐containing deodorants. Int J Cosmet Sci. 2017;39:141–8. 27506727 10.1111/ics.12358

[55] Yoshioka N , Kurata K , Takahashi T , Ariizumi M , Mori T , Fujisawa H , et al. Body odour aldehyde reduction by acetic acid bacterial extract including enzymes: alcohol dehydrogenase and aldehyde dehydrogenase. Int J Cosmet Sci. 2018;40(4):425–8. 29897105 10.1111/ics.12473

[56] Jungermann E . Antiperspirants: new trends in formulation and testing technology. J Soc Cosmet Chem. 1974;25:621–37.

[57] Bretagne A , Cotot F , Arnaud‐Roux M , Sztucki M , Cabane B , Galey J . The mechanism of eccrine sweat pore plugging by aluminium salts using microfluidics combined with small angle X‐ray scattering. Soft Matter. 2017;13(20):3812–21. 28485735 10.1039/c6sm02510b

[58] Gomes RJ , Borges M d F , Freitas Rosa M d , Castro‐Gómez RJH , Spinosa WA . Acetic acid bacteria in the food industry: systematics, characteristics and applications. Food Technol Biotechnol. 2018;56(2):139–51. 30228790 10.17113/ftb.56.02.18.5593 PMC6117990

[59] Cock IE , Wright MH , Matthews B , White A . Bioactive compounds sourced from terminalia spp. in bacterial malodour prevention: an effective alternative to chemical additives. Int J Cosmet Sci. 2019;41(5):496–508. 31381160 10.1111/ics.12567

[60] Cock IE . The medicinal properties and phytochemistry of plants of the genus terminalia (Combretaceae). Inflammopharmacology. 2015;23:203–29. 26226895 10.1007/s10787-015-0246-z

[61] Aldred EM , Buck C , Vall K . Chapter 21 – phenols. Pharmacology. London: Churchill Livingstone; 2009. p. 149–66.

[62] Suzuki ÉY , Soldati PP , Graças CM d AM , Raposo NRB . Essential oil from *Origanum vulgare* Linnaeus: an alternative against microorganisms responsible for bad perspiration odour. J Young Pharm. 2014;7(1):12–20.

[63] Ríos J‐L . Essential oils: what they are and how the terms are used and defined. Essential oils in food preservation, flavor and safety. Cambridge, Massachusetts: Academic Press; 2016. p. 3–10.

[64] Askun T , Tümen G , Satil F , Karaarslan D . Active constituents of some Satureja L. species and their biological activities. Afr J Microbiol Res. 2011;6(22):4623–33.

[65] Esmaeili A , Asgari A . In vitro release and biological activities of *Carum copticum* essential oil (CEO) loaded chitosan nanoparticles. Int J Biol Macromol. 2015;81:283–90. 26257380 10.1016/j.ijbiomac.2015.08.010

[66] Singh G , Kapoor IPS , Pandey SK , Singh UK , Singh RK . Studies on essential oils: part 10; antibacterial activity of volatile oils of some spices. Phytother Res. 2002;16(7):680–2. 12410554 10.1002/ptr.951

[67] Boskabady MH , Alitaneh S , Alavinezhad A . *Carum copticum* L.: a herbal medicine with various pharmacological effects. Biomed Res Int. 2014;2014:1–11. 10.1155/2014/569087 PMC4096002 25089273

[68] Gregoriou S , Campanati A , Rigopoulos D , Offidani AM , Stratigos A , Kontochristoulos G . Investigational topical anticholinergics in clinical development for the treatment of hyperhidrosis. Expert Opin Investig Drugs. 2021;30(5):479–82. 10.1080/13543784.2021.1900114 33691553

[69] Roberts DW , Mekenyan OG , Dimitrov SD , Dimitrova GD . What determines skin sensitization potency–myths, maybes and realities. Part 1. The 500 molecular weight cut‐off. Contact Dermatitis. 2013;68(1):32–41. 22924443 10.1111/j.1600-0536.2012.02160.x

[70] Guzman M , Dille J , Godet S . Synthesis and antibacterial activity of silver nanoparticles against gram‐positive and gram‐negative bacteria. Nanomed Nanotechnol Biol Med. 2012;8(1):37–45. 10.1016/j.nano.2011.05.007 21703988

[71] Feng QL , Wu J , Chen GQ , Cui FZ , Kim TN , Kim JO . A mechanistic study of the antibacterial effect of silverions on *Escherichia coli* and *Staphylococcus aureus* . J Biomed Mater Res. 2000;52(4):662–8. 11033548 10.1002/1097-4636(20001215)52:4<662::aid-jbm10>3.0.co;2-3

[72] Bellarin B , Mignani A , Mogavero F , Gabbanini S , Morigi M . Hybrid material based on ZnAl hydrotalcite and silver nanoparticles for deodorant formulation. Appl Clay Sci. 2015;114:303–8.

[73] Artain FS , Reader A , Fisher M , Park B , Kemp M , Johnstone J , et al. Nanotechnology and its application to medical hygiene textiles. Textiles for hygiene and infection control. Sawston: Woodhead Publishing; 2011. p. 14–26.

[74] Lolla VY , Shukla P , Jones SD , Boreyko JB . Evaporation‐induced clogging of an artificial sweat duct. ACS Appl Mater Interfaces. 2020;12(47):53403–8. 33191727 10.1021/acsami.0c13493

[75] Hu Y , Converse C , Lyons MC , Hsu WH . Neural control of sweat secretion: a review. Br J Dermatol. 2018;178(6):1233–4. 28714085 10.1111/bjd.15808

[76] Kobielak K , Kandyba E , Leung Y . Skin and skin appendage regeneration. Translational regenerative medicine. Amsterdam: Elsevier Inc.; 2005. p. 269–92.

[77] Tripathi SR , Miyata E , Ishai PB , Kawase K . Morphology of human sweat ducts observed by optical coherence tomography and their frequency of resonance in the terahertz frequency region. Sci Rep. 2015;5:1–7. 10.1038/srep09071 PMC4357862 25766116

[78] Natsch A , Emter R . The specific biochemistry of human axilla odour formation viewed in an evolutionary context. Philos Trans R Soc B: Biol Sci. 2020;375(1800):1–13. 10.1098/rstb.2019.0269 PMC7209930 32306870

[79] Chabicovsky M , Winkler S , Soeberdt M , Kilic A , Masur C , Abels C . Pharmacology, toxicology and clinical safety of glycopyrrolate. Toxicol Appl Pharmacol. 2019;370:154–69. 30905688 10.1016/j.taap.2019.03.016

[80] Paik J . Sofpironium bromide: first approval. Drugs. 2020;80:1981–6. 33236266 10.1007/s40265-020-01438-1

